# Perinatal hepatitis B virus transmission in Lao PDR: A prospective cohort study

**DOI:** 10.1371/journal.pone.0215011

**Published:** 2019-04-24

**Authors:** Vatthanaphone Latthaphasavang, Philippe Vanhems, Nicole Ngo-Giang-Huong, Philavanh Sibounlang, Phimpha Paboriboune, Laurent Malato, Valy Keoluangkhot, Syvilay Thammasack, Nicolas Salvadori, Woottichai Khamduang, Nicolas Steenkeste, Christian Trépo, Paul Dény, Gonzague Jourdain

**Affiliations:** 1 Mahosot Hospital, Xiengneun village, Sisatanak district, Vientiane capital, Lao PDR; 2 Claude Bernard University Lyon, Villeurbanne, France; 3 Emerging Pathogens Laboratory, Fondation Mérieux, Centre International de Recherche en Infectiologie, INSERM U1111, CNRS UMR5308, ENS de Lyon, UCBL1, Lyon, France; 4 Infection Control and Epidemiology Department, Hospices Civils de Lyon, Lyon, France; 5 Institut de recherche pour le développement (IRD, France), PHPT, Changklan, Muang, Chiang Mai, Thailand; 6 Chiang Mai University, Faculty of Associated Medical Sciences, Sripoom, Chiang Mai, Thailand; 7 Department of Immunology and Infectious Diseases, Harvard T.H. Chan School of Public Health, Boston, MA, United States of America; 8 Center Infectiology Lao Christophe-Mérieux, Kaoyod village, Sisatanak district, Vientiane capital, Lao PDR; 9 Fondation Mérieux, Lyon, France; 10 INSERM U_1052 UMR CNRS 5286 –Cancer Research center of Lyon, Lyon, France; 11 Université Paris 13, UFR Santé Médecine Biologie Humaine, Groupe des Hôpitaux, Paris, France; 12 Groupe des Hôpitaux Universitaire de Paris Seine Saint Denis, Service de Microbiologie, Paris, France; CEA, FRANCE

## Abstract

**Background:**

Mother-to-child transmission of hepatitis B virus (HBV) is the main cause of new infections worldwide. We aimed at assessing the percentage of infants successfully immunized in two major hospitals in Vientiane, Lao PDR where HB immune globulin (HBIg) is not available.

**Methods:**

We studied a prospective cohort of chronically HBV infected pregnant women and their infants until 6 months post-partum from January 2015 to March 2017. All infants received HB vaccine at birth and 6, 10 and 14 weeks thereafter, and HBV status was assessed at 6 months of age. HBV surface gene sequencing was performed in infected mother-infant pairs.

**Results:**

Of 153 mothers with HB surface antigen (HBsAg), 60 (39%) had detectable serum HBe antigen (HBeAg). HBeAg positive pregnant women were younger than those negative (median age 26 versus 28 years; p = 0.02) and had a significantly higher HBV viral load at delivery (median 8.0 versus 4.0 log_10_ IU/mL, p <0.001). Among the 120 infants assessed at 6 months of age, 5 (4%) were positive for HBsAg and had detectable HBV viral load by polymerase chain reaction. All were born to mothers with HBeAg and viral load >8.5 log_10_ IU/mL. However, only four (3.3%, 95% CI 0.5% to 7.0%) had a virus strain closely related to their mother’s strain. HBV surface gene mutations were detected in 4 of the 5 infected infants. Anti-HBs antibody levels were below 10 IU/L in 10 (9%) uninfected infants at 6 months of age.

**Conclusions:**

Mother-to-child transmission occurred less frequently than expected without the use of HBIg. Adding HBIg and/or maternal antiviral prophylaxis may have prevented some of these infections. The observation of unsatisfactory levels of anti-HBs antibodies in 9% of the uninfected infants at 6 months highlights the need for improvement of the universal immunization procedures.

## Introduction

An estimated 257 million people are chronically infected with hepatitis B virus (HBV) worldwide [1]. Mother-to-child transmission accounts for the majority of new chronic HBV carriers, especially in Asia. HBV can be transmitted *in utero*, during delivery or during infancy and later [2–4]. About 80–90% of infants infected at birth will develop chronic HBV infection, and 20–30% of adults who are HBV-chronically infected will experience serious complications including liver fibrosis, cirrhosis, hepatocellular carcinoma (HCC) and liver-related death [1]. HBV Genotypes B and C are common in South-East Asia. Perinatal exposure plays a key role in viral transmission [5]. Combination of hepatitis B immunoglobin at birth and vaccine is highly effective: with this strategy, several studies have observed that more than 85% of infants born to HBV-infected mothers were not infected with HBV [6–12].

Liver cancer incidence in Lao People’s Democratic Republic (Lao PDR) is one of the highest in the world in relation with a high incidence of cholangiocarcinoma and a high prevalence of HCC linked to chronic HBV infection [13]. In pregnant women presenting for antenatal care, HBV prevalence estimates have ranged between 2.9% and 8.2% [14–16], depending on settings. For hepatitis B (HB) immunization, the national recommendation is to administer a birth dose, ie a monovalent HB vaccine dose within 24 hours of birth (-HepB-BD), followed by a dose of pentavalent vaccine at 6, 10, and 14 weeks of age, regardless of maternal HBV status. However, studies have shown that in several areas of Lao PDR the HepB-BD is not administered to all newborns. For example, in 37 health facilities surveyed in five provinces, the HepB-BD coverage was 74% (interquartile range (IQR): 39%–97%) [17]. Furthermore, only 66% of children aged 9 to 16 months had sufficient anti-HBs antibody levels in a study conducted in two vaccination clinics located in urban areas: Luang Prabang Provincial Hospital and the Vientiane Mother and Child Central Hospital [15].

We assessed the percentage of infants successfully immunized in an observational cohort conducted in two major hospitals in Vientiane, Lao PDR, where HB immune globulin (HBIg) is not available although the administration of HBIg in addition to the vaccine can halve the risk of transmission according to Lee and coll.’s meta-analysis of 10 studies [18]

## Materials and methods

### Participants and clinical follow up

The cohort study was implemented at the antenatal care clinic of Mahosot Hospital, Vientiane from January 1, 2015, and then at the Vientiane Mother and Newborn Hospital from April 9, 2015. At these clinics, pregnant women are routinely screened for both Human Immunodeficiency Virus (HIV) and HBV infections at their first prenatal visit.

All pregnant women with an HBsAg positive test were informed of the study at the second antenatal care visit and invited to participate with their future infants. The inclusion criteria were: pregnancy, age ≥18 years, confirmed HBsAg positivity, and agreeing and able to bring the future infant at the planned follow up visits until 6 months after delivery. We excluded those women who had difficulties to bring their infants for follow-up. If they agreed, they were requested to provide a formal written consent. Age, education level, medical history, receipt of concomitant treatments and HBV infection status of husband/partner and other family members were recorded.

Maternal study visits were scheduled at enrollment, delivery and six weeks after delivery, and infant visits at birth and 6 months of age for clinical evaluation and determination of HBV infection status. All infants were to receive HB vaccine intramuscularly, at birth and at 6, 10 and 14 weeks following national guidelines for HB immunization. Each vaccination was immediately recorded in an individual card. Immunization was conducted using the vaccines offered by the national program, free of charge for the families i.e. a monovalent vaccine for the HepB-BD (Euvax B® 10 μg, LG Life Sciences Ltd. Korea) and as part of an pentavalent vaccine (Easyfive -TT ®, Panacea Biotec, India) at 6, 10 and 14 weeks of age. HB immune globulin (HBIg), which has not been registered in Lao PDR, is not available for infants born to HBV-infected mothers.

### Laboratory methods

Women approached for this study were pregnant women found HBsAg positive in the screening program (using the OnSite HBsAg rapid test by CTK Biotech, Inc, USA; sensitivity 100% and specificity 100% claimed to the manufacturer, the rapid test by Boson Biotech, Fujian, China; sensitivity and specificity unknown, or a HBsAg Elisa kit (Human GmbH, Wiesbaden, Germany) at Mahosot Hospital, and using the Boson HBsAg rapid test at the Mother and Newborn Hospital. Alanine aminotransferase (ALT) levels were measured using the HumaStar 600 automate (Human company, Germany).

Laboratory testing was centralized at the Center of Infectiology Laos Mérieux (CILM) for HBsAg (confirmation in pregnant women, and infants), HBe antigen (HBeAg), HB surface antibody (anti-HBs), and HBe antibody (anti-HBe) testing using Monolisa assays from Biorad (United States).

Nucleic acids were extracted from 550 microliters of plasma using the Arrow Viral NA kit, (DiaSorin, Ireland) and the NorDiag Arrow machine (Ireland) following manufacturer`s instructions.

HBV Deoxyribonucleic acid (DNA) load was measured using real-time PCR (real-time polymerase chain reaction) (FTD Hepatitis B DNA from Fast-Track Diagnostics, Luxembourg, detection limit < 50 UI/mL).

Partial Surface gene was amplified by polymerase chain reaction (PCR) and, if necessary, nested PCR. The first round PCR was performed using 5 ul of DNA extract, pol1M-pol2M primers and HotStarTaq master mix in a final volume of 50ul. The thermal cycling conditions included a first step of activation at 95°C for 15 min followed with 35 cycles of denaturation at 94°C for 30 seconds, annealing at 55°C for 30 seconds and extension at 72°C for 1 min and a final extension at 72°C for 10 minutes. The size of amplicons obtained was 1011 bp.

The second round PCR was performed with 5 ul of the first-round PCR products and Pol3M and Pol4M primers in a final volume of 50uL. The thermal cycling conditions included a first step of activation at 95°C for 15 min then 35 cycles of denaturation at 94°C for 30 seconds, annealing at 55°C for 30 seconds and extension at 72°C for 1 min then and a final extension at 72°C for 10 minutes. The amplicon size was 808 bp.

Amplified products were separated by 1% agarose gel electrophoresis and visualized with GelRed. Purification of amplicons was performed using the QIAquick PCR purification kit (Hilden, Germany).

Purified first-round amplicons were sequenced using with pol1M, pol2M, B1 and B2 and second-round amplicons with Pol3M or Pol4M primers as follows: 1 min at 96°C then 30 cycles of denaturation at 96°C for 10 seconds, annealing at 50°C for 5 seconds and extension/termination at 60°C for 4 min. Sequencing products were analyzed on an ABI 3100 genetic analyzer (Applied Biosystems, USA).

Sequences were aligned using the ClustalW multiple alignment method available within the Molecular Evolutionary Genetics Analysis (MEGA) 7 version 7.0.26. Phylogenetic analysis was conducted using the Neighbor-Joining method and tree was constructed from 16 HBV reference sequences (genotype A-H) and the sequences of the 5 infected infants and their mothers’ viruses. Phylogenetic groups robustness was evaluated through 1,000 bootstrap replicates.

Analyses of evolutionary divergence between sequences were conducted using the Maximum Composite Likelihood model [19] with MEGA7.

### Statistical considerations

This was an observational cohort to study factors possibly associated with infant HBV immunization failure, defined as either a positive HBsAg test or an anti-HBs antibody level <10 IU/L [20] at 6 months of age.

The sample size was calculated to provide enough power to detect an association between a risk factor such as HBeAg positive test and the risk of infection. Assuming a difference of at least 20% between a 25% transmission rate from HBeAg positive women and a 5% transmission rate from HBeAg negative women [6] and a 45% proportion of HBeAg positive pregnant women among those HBsAg positive [21], a total of 151 mother-infant pairs was needed to provide at least 93% power to detect a significant difference, using a two-sided alpha of 0.05. Taking into account that 5% of the infants may not be available at 6 months, 71 HBeAg positive and 88 HBeAg negative pregnant women had to be enrolled, i.e. 159 women overall.

Continuous data were summarized by medians and interquartile ranges (IQR), and categorical data by frequencies and percentages. The 95% confidence intervals (CI) of the proportions were calculated using the Clopper-Pearson method. The association between maternal and infant characteristics and infant HBV infection or immunoprophylaxis failure was assessed using Fisher’s exact test for categorical variables and Wilcoxon-Mann-Whitney test for continuous variables. It was planned to conduct multivariate analyses only if the number of events of interest was sufficient. All tests were two-sided and p < 0.05 was considered statistically significant. Data were analyzed using Stata/SE 13.0.

### Ethical considerations

The study was approved on 25 December 2014 by the National Ethic Committee for Health Research, Vientiane, Lao PDR (Study Number: 055 NIOPH/NECHR).

## Results

### Study participants at enrollment and follow up

From January 1, 2015 to March 3, 2017, 160 women with a first HBsAg positive test were enrolled. However, the positive HBsAg result was not confirmed in 7 of those 115 tested with a rapid test. Table 1 provides the characteristics of the remaining 153 participants. The women’s median age (IQR) was 28 years (24 to 30) and gestational age 25 weeks (19 to 31). None had symptoms of liver disease. Of the 153 women, 106 (69%) were unaware of their HBV infection, and 116 (76%) did not know their husband’s HBV status.

A total of 60 women (39%) had a positive HBeAg test. They were younger than those with a negative HBeAg test: median age 26 versus 28 years (p = 0.02). One HIV co-infected woman was receiving tenofovir disoproxil fumarate, lamivudine and lopinavir/ritonavir to treat her HIV infection, and two women previously received arachidonic acid, a treatment with unproven efficacy on HBV. The median ALT level was not significantly different according to HBeAg status: 23 U/L in the HBeAg-positive pregnant women versus 19 U/L in those negative (p = 0.13) (Table 1).

**Table 1:** Socio-demographic characteristics of the participants according to maternal HBeAg status.

Variables	Total	Negative HBeAg women	Positive HBeAg women	p-value
Women at baseline				
No. of women with data	153	93 ^a^	60 ^a^	
Age—years—Median (IQR)	28 (24 to 30)	28 (25 to 31)	26 (23 to 29)	0.02
Parity ^b^ Median (IQR)	2 (1 to 3)	2 (1 to 3)	2 (1 to 3)	0.75
Gestational age—weeks				
Median (IQR)	25 (19 to 31)	24 (18 to 30)	27 (19 to 32)	0.32
History of abortion—no. (%)	63 (41)	39 (42)	24 (40)	0.87
HBV infection known before pregnancy —no. (%)	47 (31)	26 (28)	21 (35)	0.38
Family income—USD/month				
Median (IQR)	450 (375 to 625)	450 (375 to 625)	450 (312 to 625)	0.67
Having family members infected with HBV—no. (%)	45 (29)	28 (30)	17 (28)	0.69
Who died of liver cancer	10/45 (22)	5/28 (18)	5/17 (29)	0.50
Partner’s age—years—Median (IQR)	31 (28 to 34)	31 (29 to 34)	31 (28 to 34)	0.62
Partner’s HBV status—no. (%)				0.69
HBsAg positive	15 (10)	9 (10)	6 (10)	
HBsAg negative	21 (14)	11 (12)	10 (17)	
Does not know	117 (76)	73 (78)	44 (73)	
ALT—**U/L—**Median (IQR)	21 (16 to 27)	19 (15 to 27)	23 (17 to 28)	0.13
**Maternal characteristics at delivery**				
No. of women with data	140	84	56	
Mode of delivery—no. (%)				0.67
Vaginal	111 (79)	68 (81)	43 (77)	
Cesarean	29 (21)	16 (19)	13 (23)	
Gestational age—weeks				
No. of women with data	130	76	54	
Median (IQR)	39 (38 to 40)	39 (38 to 40)	39 (39 to 40)	0.34
Gestational age <37 weeks—no. (%)	10 (8)	6 (8)	4 (7)	1
Complications at delivery ^c^ —no. (%)	12 (8)	5 (6)	7 (12)	0.22
ALT—U/L				
No. of women with data	120	73	47	
Median (IQR)	16 (13 to 18)	16 (14 to 18)	15 (12 to 16)	0.20
HBV DNA load (log_10_ IU/mL) at delivery or at inclusion				
No. of women with data	153	93	60	
Median (IQR)	4.90 (3.40 to 8.00)	4.00 (3.20 to 4.90)	8.00 (7.25 to 8.75)	<0.001
>5.3 log_10_ IU/mL (>200,000 IU/ml)	65 (42)	14 (15)	51 (85)	<0.001
**Maternal characteristics 6 weeks after delivery**				
No. of women with data	100	57	43	
ALT—U/L—Median (IQR)	15 (13 to 19)	14 (12 to 19)	15 (13 to 19)	0.70
**Infant characteristics**				
No. of infant with data	140	84	56	
Sex—no. (%)				0.08
Female	72 (51)	38 (45)	34 (61)	
Male	68 (49)	46 (55)	22 (39)	
Weight—grams—Median (IQR)	3200 (3000–3400)	3200 (3000–3500)	3150 (2900–3400)	0.86
**Infant HB infection and seroprotection at 6 months of age**				
No. of infant with data	120	69	51	
Positive HBsAg—no. (%)	5 (4)	0 (0)	5 (10)	0.01
Anti-HBs antibodies ≥10 IU/L—%	105 (88)	62 (90)	43 (84)	0.41
Negative HBsAg and Anti-HBs antibodies <10 IU/L—%	10 (9)	7 (70)	3 (30)	0.02

^a^ Except otherwise specified

^b^ The current pregnancy is included

^c^ 6 prolonged rupture of membranes, 2 preeclampsia, 2 breech presentation, 1 placenta previa and 1 absence of dilatation of the cervix.

**Fig 1:**
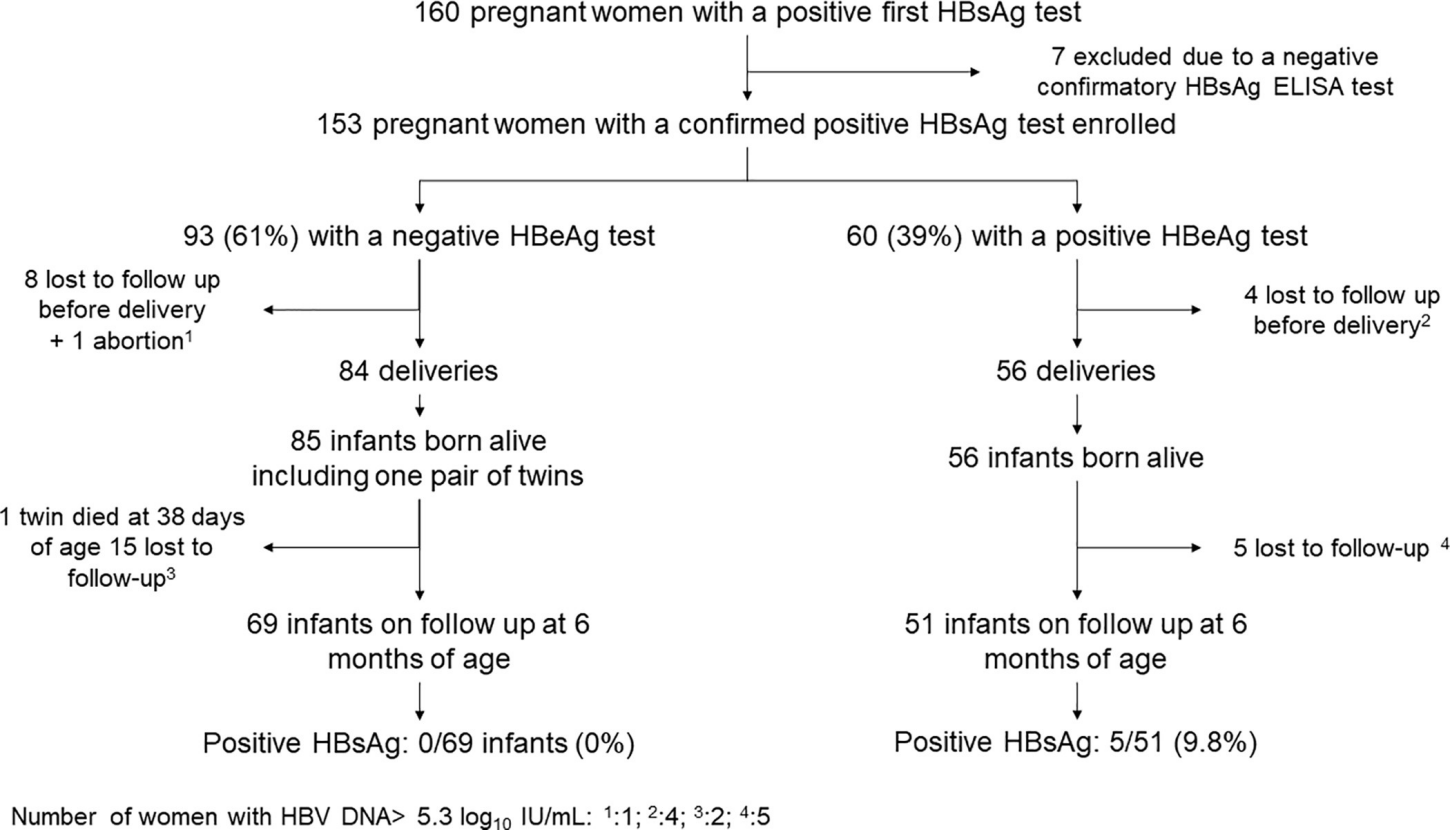
Enrollment and follow up of participants.

One woman experienced spontaneous abortion and 12 others (7.8%) were lost to follow up before delivery. Of the remaining 140 women who delivered in the study, 20 (14%) were lost to follow up with their infants by the 6-month postpartum visit. The main reason for loss to follow-up was moving to another province (see participant follow up in Fig 1). The baseline characteristics of mothers lost to follow up by 6 months postpartum were not significantly different from those who completed the study follow up.

### Characteristics at delivery and at the 6-week postpartum visit

At time of delivery, the median maternal HBV DNA load was 4.90 log_10_ IU/mL overall, 8.00 log_10_ IU/mL in HBeAg-positive mothers and 4.00 log_10_ IU/mL in HBeAg negative mothers (p <0.001). Fifty-one of the 60 HBeAg positive mothers (85%) had a viral load greater than 200,000 IU/mL (5.3 log_10_ IU/mL) compared to 14 of the 93 HBeAg negative women (15%) (p <0.001). The median ALT level was 15 U/L in HBeAg positive women and 16 U/L in those HBeAg negative (p = 0.20).

Of the 140 mothers, 12 (9%) experienced obstetrical complications: 6 prolonged rupture of membranes, 2 preeclampsia, 2 breech presentation, 1 placenta previa and 1 absence of dilatation of the cervix, with similar distribution among HBeAg positive and negative women. Also, 13 of 56 (23%) HBeAg positive and 16 of 84 (19%) HBeAg negative women delivered by cesarean section (p = 0.67), and there was a twin delivery. At the 6-week postpartum visit, the median ALT level was 15 U/L in HBeAg positive mothers and 14 U/L in those negative (p = 0.70) (Table 1). All pregnant women remained asymptomatic until 6-month postpartum.

### Vaccine administration

A total of 141 infants including a pair of twins were included in the study and information on time of vaccine administration after birth was available for 112 newborns. Of these, 110 (98%) received the HepB-BD within 24 hours of life. The median time from birth to vaccine administration was 6 hours (IQR 3 to 13), with 80 (71%) within 12 hours after birth. One newborn received vaccine 26 hours after birth because the vaccine was not available at the delivery room, and another newborn 3 days after birth because of experiencing fetal distress, which was erroneously considered as a vaccine contra-indication.

Of 120 infants seen at 6 months of age, 119 (99%) had records of HB vaccine administration at birth and at 6, 10 and 14 weeks in the Pregnancy-Infant Health Book and one infant, identified as C2 in this paper, received HB vaccine only at birth and 6 weeks of age.

### Infant HBsAg and anti-HBs

Of the 120 infants assessed at 6 months of age, five infants were found positive for HBsAg, i.e. 4.2% (95% CI 1.4% to 9.5%). Maternal age, mode of delivery, infant sex, parity, and time from birth to HepB-BD were not significantly associated with the risk of infection (Table 2).

**Table 2:** Maternal and infant characteristics according to infant HBV infection status.

Women characteristics	HBsAg negative infant	HBsAg positive infant	p-value
No. of women and infants with data available	115 ^a^	5 ^a^	
Age—years—Median (IQR)	28 (24 to 30)	26 (24 to 30)	0.71
Parity ^b^ —Median (IQR)	2 (1 to 3)	1 (1 to 3)	0.56
Mode of delivery—no. (%)			
Vaginal	92 (80)	4 (80)	1.00
Cesarean	23 (20)	1 (20)	
Complications at delivery—no.(%)	8 (7)	1 (20)	0.32
Having family members infected with HBV—no. (%)	38 (33)	0 (0)	0.21
History of abortion—no. (%)	50 (43)	2 (40)	1.00
HBV DNA load at delivery—Median (IQR)	4.60 (3.38 to 7.90)	8.90 (8.80 to 8.90)	<0.001
>5.3 log_10_ IU/mL—no. (%)	48 (42)	5 (100)	0.02
Positive HBeAg status—no. (%)	46 (40)	5 (100)	0.01
**Infants characteristics**			
Female sex—no. (%)	59 (51)	3 (60)	1.00
Median (IQR) Weight at birth—grams	3190 (2900 to 3400)	3100 (2900 to 3200)	0.77
Time from birth to HBV vaccine—hours (data available for 112 infants)	107	5	
Median (IQR)	6 (2 to 13)	16 (10 to 18)	0.12
>12 hours—no. (%)	29 (27)	3 (60)	0.14
>12 hours or no records—no. (%)	37/115 (32)	3/5 (60)	0.33

^a^ Except otherwise specified

^b^ The current pregnancy is included.

All infected infants were born to HBeAg positive mothers; thus, the estimated risk of infection was 9.8% (95% CI 3.3% to 21.4%) in infants born to HBeAg positive mothers and 0% (95% CI 0% to 5.2%) in those born to HBeAg negative mothers (p = 0.01). The mothers of the 5 infected infants had HBV DNA loads at delivery ranging from 8.6 to 9.2 log_10_ IU/mL (see Table 3 for details). Higher HBV DNA load at delivery and HBeAg positivity were associated with a higher risk of infant infection (p <0.001 and p = 0.01, respectively).

**Table 3:** Characteristics of the 5 HBV infected infants.

N	Sex	Delivery mode	Gestational age at birth (weeks)	Maternal HBeAg status	Time from birth to HBV vaccine (hours)	HBV vaccine schedule	Maternal HBV DNA (load—log_10_) at delivery	Infant HBV DNA load—log_10_ at 6 months of age	Maternal HBV genotype	Infant HBV genotype	Gene variants detected in mother at delivery	Gene variants detected in infant at 6 months of age
C1	M	Cesarean	40	Positive	3	Completed	8.90	9.23	B	B	M133I	*M133I*
C2	M	Vaginal	39	Positive	18	Uncompleted	9.20	8.40	B	B	None	*M133T*
C3	F	Vaginal	38	Positive	19	Completed	8.80	8.75	B	B	None	None
C4	F	Vaginal	39	Positive	10	Completed	8.90	7.72	C	C	None	*G145G/R*
C5	F	Vaginal	40	Positive	16	Completed	8.60	8.90	C	C	None	*G145G/A*

Anti-HBs antibodies titers measured in the serum were ≥10 IU/L in 105 infants (88%) at 6 months of age. Among the 15 with anti-HBs antibody titers <10 IU/L, 5 were positive for HBsAg. The other 10 (9%) were contacted for additional vaccine administration (Table 4).

**Table 4:** Maternal and infant characteristics according to immunoprophylaxis failure.

Women characteristics	Infant immunoprophylaxis success	Infant immunoprophylaxis failure	p-value
No. of women and infants with data available	105 ^a^	15 ^a^	
Age—years—Median (IQR)	28 (25 to 30)	27 (24 to 32)	0.84
Parity ^b^ —Median (IQR)	2 (1 to 3)	2 (1 to 2)	0.17
Mode of delivery—no. (%)			
Vaginal	83 (79)	13 (87)	0.73
Cesarean	22 (21)	2 (13)	
Complications at delivery—no. (%)	7 (7)	2 (13)	0.31
Having family members infected with HBV—no. (%)	34 (32)	4 (27)	0.80
History of abortion—no. (%)	45 (43)	7 (47)	0.79
HBV DNA load—Median (IQR)	4.60 (3.30 to 7.99)	7.30 (4.90 to 8.80)	0.03
>5.3 log_10_ IU/mL—no. (%)	43 (41)	10 (67)	0.09
Positive HBeAg status—no. (%)	43 (41)	8 (53)	0.41
**Infants characteristics**			
Female sex—no. (%)	55 (52)	7 (47)	0.78
Median (IQR) Weight at birth—grams	3200 (3000 to 3500)	3000 (2800 to 3300)	0.18
Time from birth to HBV vaccine—hours (data available for 112 infants) Median (IQR)	99	13	
Median (IQR)	5 (2 to 13)	12 (6 to 18)	0.03
>12 hours—no. (%)	26 (26)	6 (46)	0.19
>12 hours or no records—no. (%)	32/105 (30)	8/15 (53)	0.09

^a^ Except otherwise specified

^b^ Current pregnancy included.

### Origin of infant infections

**Fig 2:**
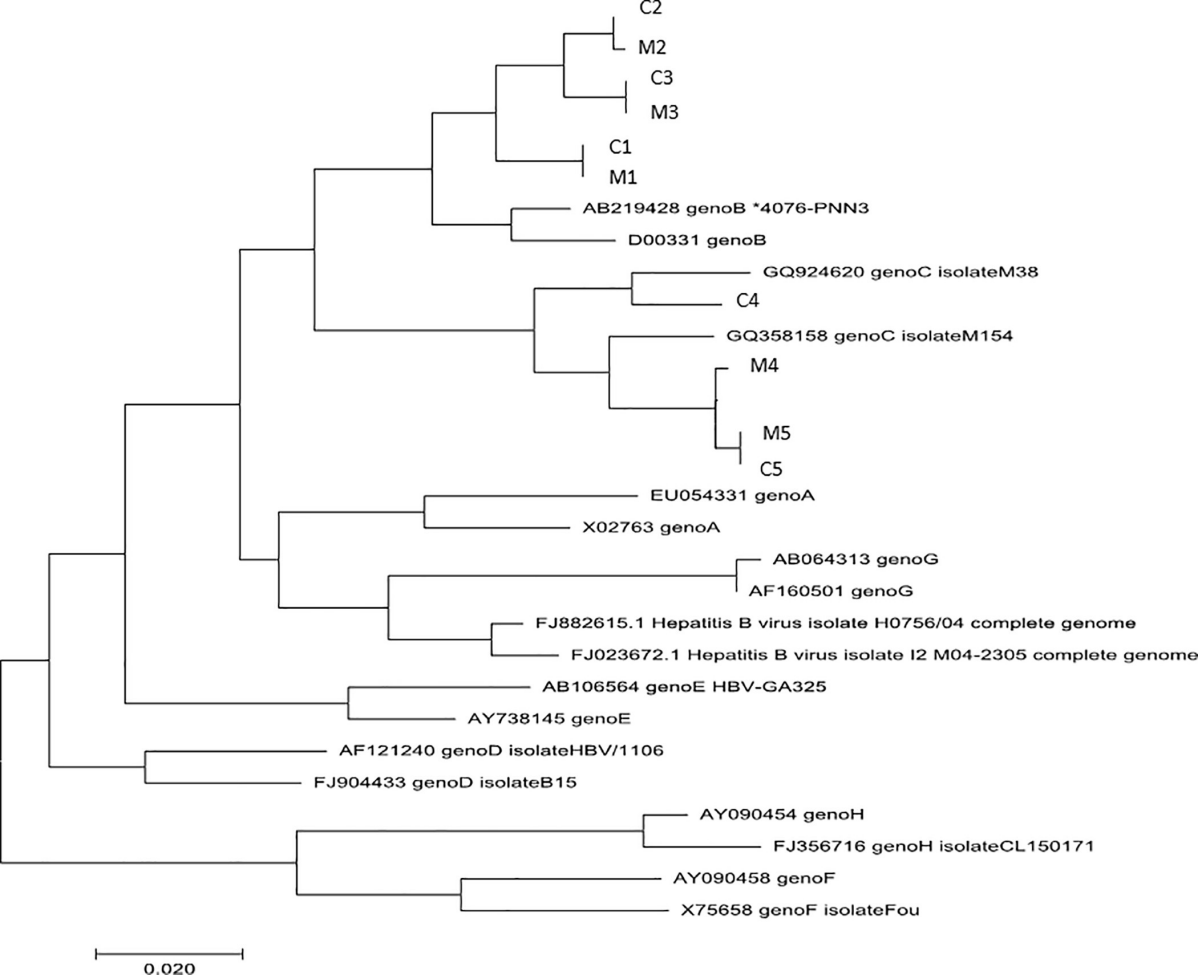
Phylogenetic tree using the Neighbor-Joining method, constructed from 16 HBV reference sequences (genotype A-H) and the sequences of the 5 infected infants and their mothers’ viruses.

The five infected infants’ relevant characteristics are summarized in Table 3. Each mother and infant were identified as M and C, respectively, followed by a number specifying the mother-infant pair. The phylogenetic analysis of HBV sequences (Fig 2) showed that four infected infants had a virus very closely related their mother’s (3.3%, 95% CI 0.5 to 7.0%) while one infant, identified as C4 in Table 3 and Fig 2, was infected by a viral strain that was not closely related to her mother’s strain although both strains were of HBV genotype C.

### Analysis of mutations in the HBsAg “a” determinant

The HBV Surface gene sequencing of the viruses of infected infants and their mothers showed no mutations in both infant and mother of pair 3; the *M133I* mutations in both the infant and mother of pair 1; and either *M133T*, *G145G/R* or *G145G/A* in the infants of the other pairs (Table 3).

## Discussion

The proportion of women with HBeAg (39%) among HBsAg positive women was similar to that reported in recent studies in Asia [11,12]. Of 141 infants born to 140 HBV chronically infected mothers in this study, 120 were assessed for HBV infection at six months of age and, of those 120 infants, 5 (4%) were found infected. As in many settings in the world, newborns had no access to HB immune globulin. As expected, all 5 infected infants were born to a mother who tested positive for HBeAg during pregnancy. The infection rate among infants born to HBeAg positive mothers was similar to that reported in most studies where infants received HBIg, i.e. from 7% to 11% [11,22–24], though two recent studies reported low rates of transmission: 2% in Thailand [12] and 4.5% in Hong Kong [25]. The relatively low risk of transmission observed in our study could be related to the short duration between birth and first administration of HB vaccine, the lack of invasive procedures, the low percentage of cesarean sections, or sampling variations.

After 6 months of age, 15 of the 120 infants (12%) (including the 5 chronically infected infants) had anti-HBs <10 UI/L. The percentage of infants (88%) successfully immunized, i.e. who achieved a protective level of antibodies at 6 months of age, seemed higher than the <66% percentage reported in two studies conducted in Laos PDR [15,26]. However, the percentage of immune prophylaxis success was low in comparison with recent clinical studies in Thailand [12] or China [22,24,27] where the percentages of uninfected infants with satisfactory antibody levels after immunization were close to 100%.

Phylogenetic analyses showed that four of the five infants had a virus strain closely related to their mother’s strain with high bootstrap values, whereas one harbored a virus strain different from her mother’s strain. This is consistent with a possible horizontal transmission and a possible facilitating role of maternal HBeAg transferred to the infant *in utero*. All but 1 of the 15 infants with unsatisfactory response to vaccine received immunization according to national recommendations. The reasons for failures are unclear. Vaccine delivery from the manufacturer to the National Vaccine Center and from this center to hospital well-baby clinics is handled by professionals specifically trained to preserve the quality of vaccines. However, we suspected that vaccine vials may have entered in close contact with frozen icepacks when delivered from the well-baby clinic to the maternity unit or during other transfers by staffs who may not be aware of the risk of freezing HB vaccine. Indeed, the World Health Organization (WHO) recommends storing HB vaccine from 2°C to 8°C as HB vaccine immunogenic proprieties are definitively lost if the vaccine has been frozen, even for a short period of time [28].

Mutations of the HBV surface gene were found in 4 of the 5 infected infants (2 with HBV genotype B and 2 genotype C): *G145G/R*, *G145G/A*, *M133T*, *M133I* (Table 3), all suspected to be associated with vaccine escape mutation [29–32].

One hundred and six (69%) mothers did not know about their HBV infection status before pregnancy and 116 (76%) pregnant women did not know about their husband’s HBV status. This supports introduction of screening in the general population as recommended by WHO for countries with high HBV prevalence [33].

A limitation was that our study was conducted in an urban area and the results may not reflect the situation nationwide. Indeed, for various reasons, including affordability of antenatal care and distance from health care facilities, a significant proportion of pregnant women do not deliver in health care facilities. Due to Ethics Committee requirements, only adult women (more than 18 years) were included, and the rate of transmission from younger women may have been different if there had been enrolled. Another limitation was the low number of infected infants limiting the possibility to conduct multivariable analyses to identify risk factors for transmission other than maternal HBeAg. The proportion of loss to follow up notified was high but the comparison of the baseline characteristics of those lost to follow up and the others did not indicate differences.

## Conclusion

In this first study describing HBV perinatal transmission in Lao PDR, we observed a relatively low rate of MTCT considering the lack of HBIg. Maternal antiviral treatment may have prevented four infections acquired from the mothers. However, one infant was infected by a virus unrelated to her mother’s virus, strongly suggesting immunization failure, and 10 uninfected infants had unsatisfactory levels of anti-HBs antibodies at 6 months. This supports the need for more intensive measures to prevent mother-to-child transmission through the use of immune globulin and/or maternal antiviral prophylaxis, but also the need for improvement of the universal immunization procedures.

## Supporting information

S1 File Data in S3-Final HBV Pregnancy-PLoS ONE. (XLSX)
